# Data relating to the transcriptomes of human lung epithelial cells exposed to radon-emitting rock, tobacco smoke or cannabis smoke

**DOI:** 10.1016/j.dib.2018.11.009

**Published:** 2018-11-05

**Authors:** Julie J. Loiselle, Jose M. Knee, Leslie C. Sutherland

**Affiliations:** a Health Sciences North Research Institute, Sudbury, ON, Canada P3E 2H3; b Biomolecular Sciences Program, Laurentian University, Sudbury, ON, Canada P3E 2C6

## Abstract

Presented herein are RNA expression data linked to the exposure of human lung epithelial cells to either low dose radon-emitting rock, tobacco smoke or cannabis smoke. Two cell lines were used, one representing a ‘normal’ lung epithelial cell (BEAS-2B, derived from immortilized bronchial epithelial cells from a cadaver) and one representing a ‘cancerous’ lung epithelial cell (NCI-H1975, derived from a primary lung adenocarcinoma from a non-smoker). Control cells were cultured under standard conditions. Test cells were either (a) continuously cultured in the presence of pulverized uranium-containing rock emitting 38 Bq/m^3^ radon, or (b) exposed five days a week, to a 1:10,000 dilution of either tobacco or cannabis smoke from one cigarette. RNA was extracted from the cells at various time-points over a period of 1–17 weeks (7–140 days). cDNA libraries were prepared from the RNA, and the libraries were sequenced. Raw, aligned sequencing data, from 38 biosamples, are available through a public repository. Differential gene expression data, relating to control and test samples from various time-points, are linked to this article. Detailed analyses relating to these data can be found in the article “Human lung epithelial cells cultured in the presence of radon-emitting rock experience gene expression changes similar to those associated with tobacco smoke exposure” [1].

**Specifications table**

**Table:** 

Subject area	Biology
More specific subject area	Human cell line gene expression
Type of data	.bam sequence files, through the NCBI URL
	.Excel differential gene expression files, linked to this article
How data was acquired	Illumina NextSeq. 500 machine
Data format	Filtered (trimmed and aligned) – .bam files
	Analyzed (through Cufflinks) – .Excel files
Experimental factors	• RNA was extracted using Tri-Reagent (Molecular Research Center, Inc.). • Only samples with Bioanalyzer (Agilent Technologies) RNA integrity values ≥ 8.9 were used to generate cDNA libraries. • cDNA libraries were generated using a TruSeq Stranded mRNA LT Sample Prep Kit (Illumina), with fragments averaging 260–305 base-pairs. • Twelve libraries were diluted, pooled and loaded into a single flow cell from a NextSeq. 500/550 Hi-Output v2 kit (150 cycles) (Illumina). • Pair-ended reads were sequenced, with 75 nucleotides read per end. • For inclusion, each sample required a minimum of 23 million reads, with over 90% of the bases having a Q30 score. • Raw sequencing data meeting the inclusion criteria were uploaded to the Illumina BaseSpace Sequence Hub, where adaptors were removed, sequences were aligned to reference human genomes Hg19 or Hg38, and trimmed. • Differential gene expression of the aligned and trimmed data was carried out using Cufflinks (v2.2.1), with advanced settings for (a) compatible hits normalization, (b) fragment bias correction, and (c) multi-read correction. To be considered significant, expression differences, on a linear scale, of at least 2-fold between test and control samples were chosen.
Experimental features	Human lung cell lines were cultured under standard conditions of 37 °C, 5% CO_2_ with humidity. Test cells were additionally exposed to radon-emitting rock, tobacco or cannabis smoke. At specific time-points, cells were collected, RNA was extracted, and the RNA treated as described immediately above.
Data source location	Sudbury, Canada
Data accessibility	• Raw sequencing data, as .bam files, are in the NCBI public repository, under Sequence Read Archive Accession Number SRP150582 and BioProject Number PRJNA476229, https://www.ncbi.nlm.nih.gov/sra/SRP150582. • Analyzed data of differential gene expression, as .Excel files, are linked to this article.

**Value of the data**

• Demonstrates that human epithelial cells can experience significant gene expression changes when cultured for as little as one week in the presence of 38 Bq/m^3^ radon-emitting rock. These data could be used in an expanded analysis of a comparison of the transcriptome changes associated with both lower and higher doses of radiation in order to examine gene expression in relation to function, particularly in relation to carcinogenesis.
• Reveals a significant reversal of gene expression directionality, occurring sometime between 7 and 14 weeks of exposure to radon-emitting rock. These data suggest that the experimental set-up would be useful to study carcinogenic versus adaptive responses to radiation exposure.
• Reveals a dose-related upregulation of the aldo-keto reductase gene, *AKR1C3*, in the BEAS-2B and NCI-H1975 cells that were cultured in the presence of radon-emitting rock. These data suggest that a functional examination of *AKR1C3* expression could provide valuable insight into its potential role as a regulator of carcinogenesis.
• Reveals some of the earliest gene expression changes associated with exposure to cigarette smoke, which could be of use to researchers attempting to identify drivers of the transformation process.
• Reveals some of the earliest gene expression changes associated with exposure to cannabis smoke, which could be of use for further gene-centric functional analyses to better understand how cells respond to cannabis smoke.

## 1 Data

RNA from the control and test samples for each condition (radon, tobacco smoke and cannabis smoke) was sequenced and aligned to the human genome, a process that generated a .bam file. In total, the sequences from 38 biosamples were uploaded into the Sequence Archive Repository (https://www.ncbi.nlm.nih.gov/sra/SRP150582). Each sequence can be accessed using its own accession number (listed in Table 1), or all sequences can be accessed using the accession number SRP150582, with BioProject number PRJNA476229. Basic analysis of these data, in the form of a comparison between the control and test sample for each condition from each time-point taken, has been included with this article, as a link. A summary of all available data is contained in Table 1. To note, comparisons of gene expression between test samples from different time-points was not possible because there were significant gene expression changes over-time, within the cell populations. The excel files provide easily accessed, useful information showing how exposure to a specific condition affected gene expression, compared to the non-exposed control, at that point in time.

**Table 1:** Summary of data linked to this article.

**Cell line/exposure/time-point**	**Sequencing files accessed through**https://www.ncbi.nlm.nih.gov/sra/SRP150582**, with BioProject Number PRJNA476229, or directly via the individual URL listed below for each sequence (accession numbers 9428764–9428801)**	**Excel spread sheet indicator: containing Cufflinks comparison data between the two NCBI accession numbers indicated**
BEAS-2B/smoke control/week 2	9428764: https://www.ncbi.nlm.nih.gov/sra/9428764	
BEAS-2B/smoke control/week 8	9428765: https://www.ncbi.nlm.nih.gov/sra/9428765	
BEAS-2B/smoke control/week 12	9428766: https://www.ncbi.nlm.nih.gov/sra/9428766	
BEAS-2B/smoke control/week 16	9428767: https://www.ncbi.nlm.nih.gov/sra/9428767	
BEAS-2B/tobacco/week 2	9428768: https://www.ncbi.nlm.nih.gov/sra/9428768	2BctrlW2_vs_2BTobW2.gene_exp: 9428764 vs 9428768
BEAS-2B/tobacco/week 8	9428769: https://www.ncbi.nlm.nih.gov/sra/9428769	2BctrlW8_vs_2BTobW8.gene_exp: 9428765 vs 9428769
BEAS-2B/tobacco/week 12	9428770: https://www.ncbi.nlm.nih.gov/sra/9428770	2BctrlW12_vs_2BTobW12.gene_exp: 9428766 vs 9428770
BEAS-2B/tobacco/week 16	9428771: https://www.ncbi.nlm.nih.gov/sra/9428771	2BctrlW16_vs_2BTobW16.gene_exp: 9428767 vs 9428771
BEAS-2B/cannabis/week 2	9428772: https://www.ncbi.nlm.nih.gov/sra/9428772	2BctrlW2_vs_2BCannW2.gene_exp: 9428764 vs 9428772
BEAS-2B/cannabis/week 8	9428773: https://www.ncbi.nlm.nih.gov/sra/9428773	2BctrlW8_vs_2BCannW8.gene_exp: 9428765 vs 9428773
BEAS-2B/cannabis/week 12	9428774: https://www.ncbi.nlm.nih.gov/sra/9428774	2BctrlW12_vs_2BCannW12.gene_exp: 9428766 vs 9428774
BEAS-2B/cannabis/week 16	9428775: https://www.ncbi.nlm.nih.gov/sra/9428775	2BctrlW16_vs_2BCannW16.gene_exp: 9428767 vs 9428775
BEAS-2B/radon control/day 7	9428776: https://www.ncbi.nlm.nih.gov/sra/9428776	
BEAS-2B/radon/day 7	9428777: https://www.ncbi.nlm.nih.gov/sra/9428777	2BminRaD7_vs_2BpluRaD7.gene_exp: 9428776 vs 9428777
BEAS-2B/radon control/day 28	9428778: https://www.ncbi.nlm.nih.gov/sra/9428778	
BEAS-2B/radon/day 28	9428779: https://www.ncbi.nlm.nih.gov/sra/9428779	2BminRaD28_vs_2BpluRaD28.gene_exp: 9428778 vs 9428779
BEAS-2B/radon control/day 49	9428780: https://www.ncbi.nlm.nih.gov/sra/9428780	
BEAS-2B/radon/day 49	9428781: https://www.ncbi.nlm.nih.gov/sra/9428781	2BminRaD49_vs_2BpluRaD49.gene_exp: 9428780 vs 9428781
BEAS-2B/radon control/day 98	9428782: https://www.ncbi.nlm.nih.gov/sra/9428782	
BEAS-2B/radon/day 98	9428783: https://www.ncbi.nlm.nih.gov/sra/9428783	2BminRaD98_vs_2BpluRaD98.gene_exp: 9428782 vs 9428783
NCI-H1975/radon control/day 7	9428784: https://www.ncbi.nlm.nih.gov/sra/9428784	
NCI-H1975/radon/day 7	9428785: https://www.ncbi.nlm.nih.gov/sra/9428785	H1975minRaD7_vs_H1975pluRaD7.gene_exp: 9428784 vs 9428785
NCI-H1975/radon control/day 20	9428786: https://www.ncbi.nlm.nih.gov/sra/9428786	
NCI-H1975/radon/day 20	9428787: https://www.ncbi.nlm.nih.gov/sra/9428787	H1975minRaD20_vs_H1975pluRaD20.gene_exp: 9428786 vs 9428787
NCI-H1975/radon control/day 28	9428788: https://www.ncbi.nlm.nih.gov/sra/9428788	
NCI-H1975/radon/day 28	9428789: https://www.ncbi.nlm.nih.gov/sra/9428789	H1975minRaD28_vs_H1975pluRaD28.gene_exp: 9428788 vs 9428789
NCI-H1975/radon control/day 49	9428790: https://www.ncbi.nlm.nih.gov/sra/9428790	
NCI-H1975/radon/day 49	9428791: https://www.ncbi.nlm.nih.gov/sra/9428791	H1975minRaD49_vs_H1975pluRaD49.gene_exp: 9428790 vs 9428791
NCI-H1975/radon control/day 98 - replicate 1	9428792: https://www.ncbi.nlm.nih.gov/sra/9428792	
NCI-H1975/radon control/day 98 - replicate 2	9428793: https://www.ncbi.nlm.nih.gov/sra/9428793	
NCI-H1975/radon/day 98 - replicate 1	9428794: https://www.ncbi.nlm.nih.gov/sra/9428794	
NCI-H1975/radon/day 98 - replicate 2	9428795: https://www.ncbi.nlm.nih.gov/sra/9428795	H1975minRaD98_vs_H1975pluRaD98.gene_exp: 9428792 & 9428793 vs 9428794 & 9428795
NCI-H1975/radon control/day 119 - replicate 1	9428796: https://www.ncbi.nlm.nih.gov/sra/9428796	
NCI-H1975/radon control/day 119 - replicate 2	9428797: https://www.ncbi.nlm.nih.gov/sra/9428797	
NCI-H1975/radon/day 119 - replicate 1	9428798: https://www.ncbi.nlm.nih.gov/sra/9428798	
NCI-H1975/radon/day 119 - replicate 2	9428799: https://www.ncbi.nlm.nih.gov/sra/9428799	H1975minRaD119_vs_H1975pluRaD119.gene_exp: 9428796 & 9428797 vs 9428798 & 9428799
NCI-H1975/radon control/day 140	9428800: https://www.ncbi.nlm.nih.gov/sra/9428800	
NCI-H1975/radon/day 140	9428801: https://www.ncbi.nlm.nih.gov/sra/9428801	H1975minRaD140_vs_H1975pluRaD140.gene_exp: 9428800 vs 9428801

## 2 Experimental design, materials and methods

Control BEAS-2B and NCI-H1975 cells were cultured under standard conditions of 37 °C, 5% CO_2_ in an humidified incubator. Test BEAS-2B cells were additionally either continuously cultured in the presence of 38 Bq/m^3^ radon-emitting rock, or exposed to one cigarette per day for five days per week to a 1:10,000 dilution of either tobacco or cannabis smoke. Test NCI-H1975 cells were likewise continuously cultured in the presence of 38 Bq/m^3^ radon-emitting rock. Exposures ranged between one and seventeen weeks. Control and test cells originated from the same parental stock, and were cultured and passaged simultaneously, so that at each time-point examined, the control and test cell populations were from the same passage number. Following isolation of polyadenylated RNA from each sample collected, and sequencing of the final cDNA products, significant gene expression differences between each control and test cell population, at a particular time-point, were examined. Details relating to the experimental design, materials and methods can be found in [1].
